# Large-scale automated image analysis for computational profiling of brain tissue surrounding implanted neuroprosthetic devices using Python

**DOI:** 10.3389/fninf.2014.00039

**Published:** 2014-04-29

**Authors:** Nicolas Rey-Villamizar, Vinay Somasundar, Murad Megjhani, Yan Xu, Yanbin Lu, Raghav Padmanabhan, Kristen Trett, William Shain, Badri Roysam

**Affiliations:** ^1^BioImage Analytics Laboratory, Department of Electrical and Computer Engineering, University of HoustonHouston, TX, USA; ^2^Center for Integrative Brain Research, Seattle Children's Research InstituteSeattle, WA, USA

**Keywords:** Python, neuroprostetic device, C++, image processing software, segmentation, microglia tracing, neuroscience

## Abstract

In this article, we describe the use of Python for large-scale automated server-based bio-image analysis in FARSIGHT, a free and open-source toolkit of image analysis methods for quantitative studies of complex and dynamic tissue microenvironments imaged by modern optical microscopes, including confocal, multi-spectral, multi-photon, and time-lapse systems. The core FARSIGHT modules for image segmentation, feature extraction, tracking, and machine learning are written in C++, leveraging widely used libraries including ITK, VTK, Boost, and Qt. For solving complex image analysis tasks, these modules must be combined into scripts using Python. As a concrete example, we consider the problem of analyzing 3-D multi-spectral images of brain tissue surrounding implanted neuroprosthetic devices, acquired using high-throughput multi-spectral spinning disk step-and-repeat confocal microscopy. The resulting images typically contain 5 fluorescent channels. Each channel consists of 6000 × 10,000 × 500 voxels with 16 bits/voxel, implying image sizes exceeding 250 GB. These images must be mosaicked, pre-processed to overcome imaging artifacts, and segmented to enable cellular-scale feature extraction. The features are used to identify cell types, and perform large-scale analysis for identifying spatial distributions of specific cell types relative to the device. Python was used to build a server-based script (Dell 910 PowerEdge servers with 4 sockets/server with 10 cores each, 2 threads per core and 1TB of RAM running on Red Hat Enterprise Linux linked to a RAID 5 SAN) capable of routinely handling image datasets at this scale and performing all these processing steps in a collaborative multi-user multi-platform environment. Our Python script enables efficient data storage and movement between computers and storage servers, logs all the processing steps, and performs full multi-threaded execution of all codes, including open and closed-source third party libraries.

## 1. Introduction

Our goal is to quantify tissue perturbations inflicted by implanted neural recording devices, since their performance depends upon the state of the surrounding tissue. Our current understanding of microglia is largely based on qualitative visual analysis of two-dimensional (2-D) micrographs. There is a compelling need for an objective, quantitative, and fully 3-D analysis of microglia arbors over extended (multi-millimeter) tissue regions large enough to encompass the implanted device. Toward this goal, we present a method combining 3-D confocal imaging of extended tissue regions, large-scale computational image analysis, quantitative neuromorphology, and bio-informatics. The created processing pipeline was developed using python as the building block to join all the required modules together.

**Figure 1:**
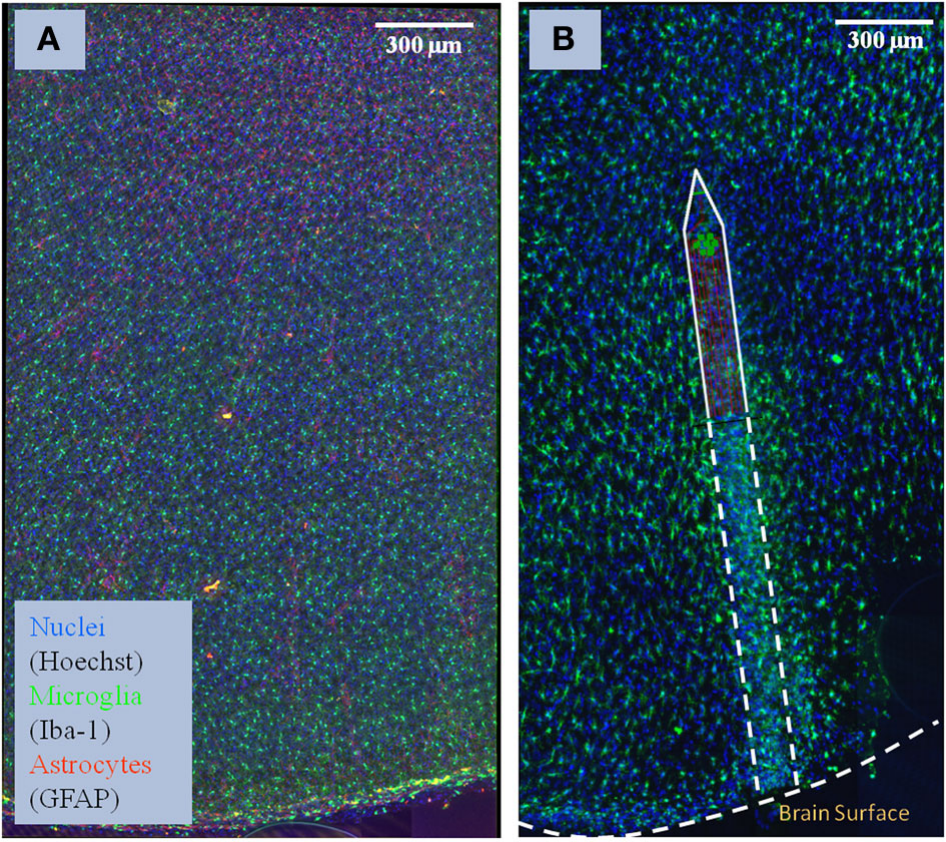
**Sample of brain tissue images used to study the impact of implanted neuro-prosthetic devices. (A)** Maximum-intensity projection of a multi-channel confocal montage of normal rat brain tissue, and **(B)** tissue after 1 month of implantation of the neuro-prosthetic device. An outline of the device is shown in the picture to demonstrate how different the tissue is near the device compared to far away from the device. Blue color represents the Nuclei channel, green represents the Microglia channel, and red represents the Astrocyte channel. This image illustrates the complexity and size of the image data required to be processed by our study.

The images consist of coronal sections of 4% paraformaldehyde fixed rat brain motor cortices, some with electrodes implanted for 30 days (NeuroNexus, Ann Arbor, MI), which were cut into 100-μm thick slices, and labeled (GFAP for astrocytes, Iba-1 for microglia, Hoechst for nuclei, and NeuroTrace for neurons). A Rolera EM-C2 camera (QImaging, Surrey, Canada) on an Olympus spinning-disk confocal microscope was used to record images (×30, 1004 × 1002 pixels at a resolution of 0.267 μm/pixel, 14 bits/pixel, step size of 0.3 m). Overlapping image tiles were combined into a 3-D montage of extended fields. Figure 1 shows examples of the two types of brain tissue which are needed to compare: normal tissue, and tissue with an implanted neuroprostetic device. In order to study the changes between the normal tissue and the tissue with the implanted device in these complex biological environments, we need to first identify the regions of interest (i.e., cells and microglia/neuron arbors) and then use appropriate mathematical descriptors to model the differences.

Solving the problem requires integration of multiple software systems, because no one particular toolkit offers all the required algorithms to process these images. In general, to routinely process these big images, a fully automatic pipeline is required that is capable of integrating all software tools (open and close source tools), developed in different programming languages (C++, Java, C, etc.), into a one-click single solution, which allows a non programming expert to process these images. In particular, FARSIGHT (Fiji Schindelin et al., 2012; Roysam, 2013) form the building block of our pipeline.

The FARSIGHT system, which is a quantitative open-source toolkit developed for studying complex and dynamic biological microenvironments from 4D/5D microscopy data, offers an extended number of different image processing algorithms. These algorithms are developed for the general purpose of analyzing different biological images. This open source toolkit includes state of art segmentation, registration, mosaicing, and tracing algorithms along with data visualization and analysis modules. ImageJ (the newer version is called Fiji) is a public domain Java-based image processing program that can be used to display, edit, analyze, process, and save many image formats including TIFF, GIF, JPEG, BMP, DICOM, FITS and “raw.” In particular, for the explained pipeline we have used the preprocessing algorithms offered by ImageJ/Fiji which are integrated into the proposed pipeline.

The proposed solution to analyze the huge amount of data by this study consists of a number of core steps: a registration and mosaicing step, followed by a preprocessing step to denoise the images, extraction of meaningful features, which are primarily based on segmentation/classification of cell nuclei, and tracing of microglia arbors. Although each of these steps can be performed manually for a given dataset, integrations of the results of each algorithm are labor intensive and prone to human errors. Furthermore, the study of these complex biological micro-environments requires collaborative work between interdisciplinary groups that requires careful maintenance of record files to keep track of the steps performed in each particular dataset. In addition, one must often process legacy datasets with certain change in parameters. One efficient way to obtain consistent and reproducible results is through the use of a pipeline like the one developed here.

Our approach addresses these problems in the following ways. First, since this pipeline is based on a pluggable architecture, each of these modules can be turned on/off, or a new module can be plugged in based on the need of a given problem. Second, given that this pipeline is designed to be used on a routine basis by biologists and other people who might know little of image processing, the careful design and organization of the modules are of utmost importance to allow re-processing of data for occasions when the default pipeline does not work. This failure to cope with a particular dataset can be due to changes in the imaging protocol, experimental condition, new artifacts introduced in the data, etc. The maintenance of the record files at each stage will help the image processing expert to fix encountered problem in a more efficient way.

In this paper, we present a processing pipeline targeted toward a biologist who can extract the relevant features for a given dataset without knowing the intricate details of the image processing algorithms. This pipeline is based on the idea of one-click processing automation: to accomplish this goal, the parameters used to tune the algorithm are maintained in a separate file that can be changed according to the requirements of each dataset. We illustrate the different steps of our method (registration, segmentation, tracing, and feature extraction) by running the pipeline on one of the datasets and present the results as well as limitations of the proposed solution. Also, improvements over the used algorithms are described, which were required in order for our pipeline to work in a realistic amount of time in such large scale.

## 2. Materials and methods

**Figure 2:**
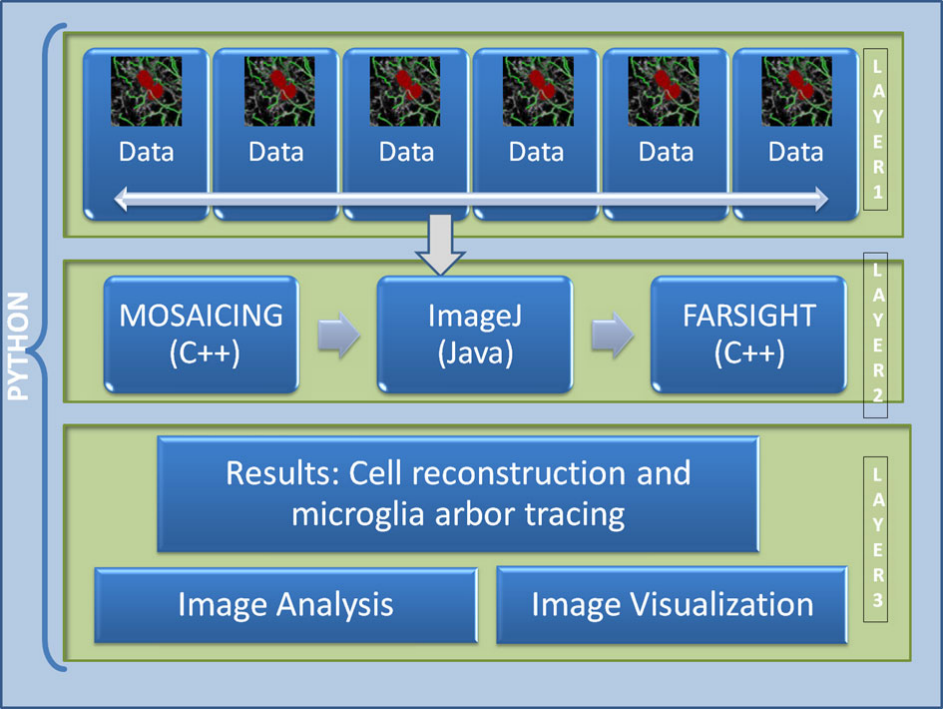
**Architecture of the proposed pipeline**. Layer 1 is the data layer which consist of the overlapping tiles acquired by the motorized microscope, Layer 2 consists of all the image processing and feature extraction algorithms, Layer 3 is the result visualization and analysis layer.

**Figure 3:**
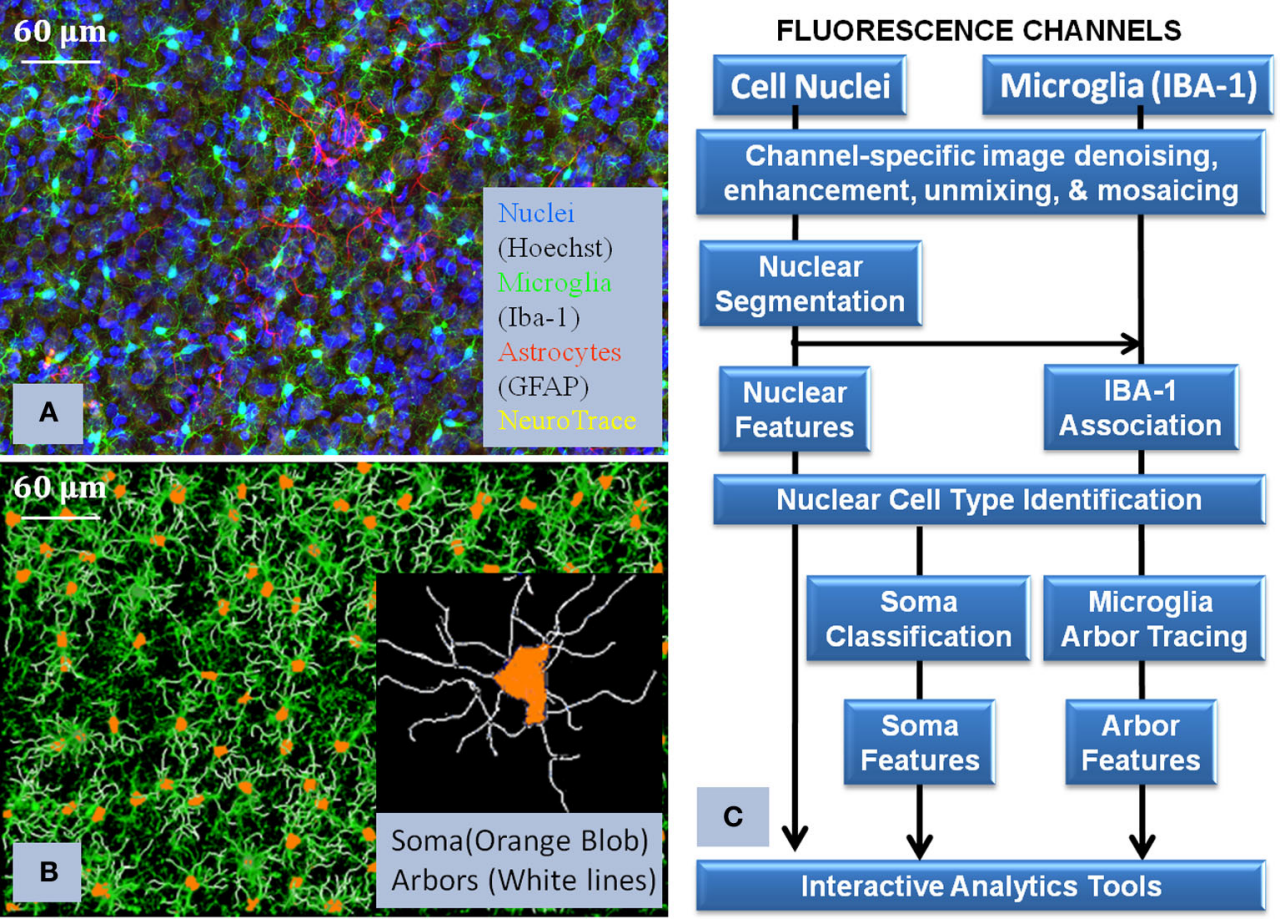
**Illustrates the core processing modules integrated in the pipline (Layer 2 of Figure 2), together with the visualization of the raw data and the corresponding reconstruction. (A)** shows the multichannel raw data, **(B)** shows the flow chart of how the algorithms were interconnected in order to process the images, and **(C)** shows the final reconstruction of the microglia and its corresponding processes. This flowchart illustrates the complexity of the required solution and how we approach the problem to successfully use Python to integrate all the modules.

The current pipeline was developed to create a modular architecture that integrates different algorithms written in distinct programming languages capable of analyzing high-content confocal images of brain tissue, with the goal of studying the immune system reaction to the implantation of neuro-prosthetic devices. The architecture of the pipeline is presented in Figure 2. The first layer consists of the raw data, which in our case, is in the range of 100–200 GB per channel (each dataset consist of 4 channels: microglia, neurons, cell nuclei, and astrosyte). In general, the core algorithms used by our pipeline require a memory usage of about 4–10 times the size of the input image; for example, in our case, the tracing algorithm requires about 8 times the size of the microglia channel. To process this amount of data is challenging even for today's state of the art processors. To circumvent this problem we have developed a robust architecture that is invariant to the size of the input data. Our approach is based on the divide-and-conquer design paradigm. The proposed pipeline can run using multiple cores on a fixed size image data (called dice), which can be specified by the user according to the system configuration. This approach allows the user to process a 100 GB image in a system with a limited memory such as an 8 GB RAM by restricting the dice size. We have found that this simple method with a carefully designed merge strategy, is powerful enough to deal with the problem at hand. The second layer consists of the core image processing algorithms which are mainly developed in different programming languages like Java, C++, and C, including open and closed-source third party libraries. The current architecture allows the modules to be easily turned on or off according to user needs. Since this layer is based on a pluggable architecture, the user can design a particular pipeline that suits their requirement or the user can use the default pipeline as described in Figure 3. The third layer merges the results from each dice in coherent way. The final results can be integrated with any image analysis and visualization tools; in our case, we have used the tools provided by FARSIGHT to analyze and display the results.

### 2.1. Mosaic and registration

This application requires the correct registration and mosaicing of high-resolution three-dimensional (3-D) images of extended tissue regions that are much wider than the lateral field of view of the microscope. To accomplish that, a series of partial views of the overall region of interest are acquired, and then combined to form a synthetic image (i.e., mosaic or montage). Tsai et al. (2011) developed a fully automatic and efficient registration and mosaicing approach which was included in FARSIGHT. This algorithm consists of three main steps. First, a pair wise image registration is computed between adjacent image tiles. In order to avoid massive computational cost, and given that the image acquisition set-up obtains a series of images by shifting the stage, the spatial transformation from one image tile to the next is largely accounted by the lateral shift. For this, the maximum-intensity axial projection is registered at a low computational cost. This is accomplished by using the generalized dual-bootstrap iterative-closest-poling (GDB-ICP) algorithm (Yang et al., 2007). Subsequently a 3-D transformation is performed using the Insight Toolkit (Ibanez et al., 2003). This algorithm performs a regular-step gradient descent minimization of the inverse pixel-wise normalized cross-correlation error. Second, a globally consistent joint registration procedure is performed. This step is required since pair-wise registration of tiles can introduce inconsistency in some regions of the montage. These errors have a magnified impact with the increase in the size of the montage. The final step consists on creating the montage from the obtained transformations. This part of the algorithm, as developed by the original authors, requires considerable amount of time and memory. We have improved this part of the algorithm by a careful design of the region of interest which is required to be stitched together, and by doing so, the time to montage was reduced by a factor of approximately 20 and the memory requirement by a factor of 2. In a nutshell, what we have developed is a way to register specific image regions and avoid the unnecessary creation of multiple copies of the original image. Also, by using the information present in the transformation parameters, we can create a bounding box containing the appropriate image space and in this way the memory usage is reduced considerably. This also allows the process to be run in parallel. Given that the typical image size per channel is about 300 GB, this improvement is of significant importance to make the algorithm practical. We have also extended this algorithm to the state-of-the-art microscopy images consisting of 14-bit/pixel. In this part of our pipeline, python allows us to create a combined solution which consists of the union of an open-source and closed-source algorithm into a single framework which is fully automatic.

### 2.2. Image preprocessing

For our application we have found the combination of different algorithm which works best for us. We have used the Insight Toolkit (Ibanez et al., 2003) for performing median filtering in the images. This is a classic technique used for noise reduction. The next step consists of illumination correction, for which different approaches such as the classic top-hat filtering technique were tested. However, we found that using the rolling-ball filtering algorithm included in ImageJ/Fiji gives the best trade-off between time and accuracy of the results. Python was used as a tool to integrate these two preprocessing algorithms into a single framework. The way in which our pipeline was written allowed us to easily integrate other preprocessing steps according to the problem requirement. In some cases, particularly challenging areas of the image required additional preprocessing steps. For this, we have provided ways, in particular the concept of regions of interests, to apply different processing algorithms to different parts of images. This is especially required for images with non uniform staining due to higher concentration of microglial cells near the device.

### 2.3. Curvelets

The accuracy of the automated tracing algorithms is limited by the image quality i.e., signal-to-noise ratio, contrast, and image variability. In particular, in order to deal with the discontinuities in the arbors of the microglia, these images need to be preprocessed by a suitable algorithm that can preserve and enhance the curvilinear structure of the arbors, close the gaps and at the same improve the signal-to-noise ratio. We also need algorithms that are scalable and fast to deal with the high throughput images like the ones described in this study whose size vary from tens to hundreds of gigabytes.

Recently, a number of geometric transforms based on the concept of multi-scale wavelet transform have emerged such as curvelets (Candes et al., 2006), ridgelets (Candes et al., 2006) and more generally, shapelets (Kelly and McKay, 2004). These techniques are inherently multi-scale and do not require the extent of scales to be explicitly and tightly specified unlike the Hessian based ridge (Meijering et al., 2003) detector to estimate local direction. The curvelet transform is particularly suitable for handling microglia images since the structures of interest are curvilinear. This transform not only provides a shape specific methodology for image denoising and rejection of non-curvilinear structures (e.g., cell nuclei and various image artifacts), but also provides estimates of local structure orientation at each voxel (i.e., a dense orientation map).

Curvelets are two dimensional waveforms that can be used for sparse representation of curvilinear structures in images. In space, a curvelet Ψ^*D*^_*j,l,k*_ at scale *j* is an oriented needle whose effective support is a 2^*j*^ by 2^*j*/2^ rectangle and thus obeys the parabolic scaling relation width ≈ 2×length. In frequency, a curvelet at scale *j* is a wedge whose frequency support is again inside a rectangle, but of 2^*j*^ by 2^*j*/2^. Unlike wavelets, curvelets are localized not only in position (the spatial domain) and scale (the frequency domain), but also in orientation. In our work, we use the fast discrete curvelet transform implementation to enhance the arbors of microglia and compute their local orientation at each pixel. The curvelet coefficients are simply the inner product of the input with each of the basis of curvelet waveforms. For example, given a digital pixel array [*t*_1_, *t*_2_] ∈ *L*^2^, 0 < *t*_1_, *t*_2_ < *n*, the digital curvelet coefficient *C*^*D*^(*j, k, l*) can be computed as

(1) $$C^{D}\left(i,k,l\right)=\sum_{0⩽t_{1},t_{2}⩽n}f\left[t_{1},t_{2}\right]\Psi_{i,j,k}^{D}\left[t_{1},t_{2}\right],$$

where Ψ^*D*^_*i,l,k*_ is the digital (*D*) curvelet wave form, *j* is the scale parameter, *l* refers to the orientation parameter and *k* = (*k*_1_, *k*_2_) refers to the spatial location parameter of the curvelet waveform. One approach to image enhancement is to use a threshold to eliminate small curvelet coefficients and retain only the large ones. If *C*^*D*^_*t*_(*j, k, l*) denotes the coefficients after enhancements and *T*(*i,j*) the threshold, then

(2) $$C_{t}^{D}\left(i,k,l\right)=\left\{C^{D}\left(j,k,l\right)|C^{D}\left(j,k,l\right)>T\left(i,j\right)\right\}.$$

Then, the enhanced image can be obtained by taking the inverse curvelet transform of *C*^*D*^_*t*_. Due to the memory requirement and compatibility with the FARSIGHT framework, we have used a curvelet tiling approach which we have developed using c++, and integrated in the main pipeline by using python.

### 2.4. Segmentation and classification

In order to perform the analysis of an image, the first and most important step is to identify regions corresponding to individual cells (cell segmentation) to extract meaningful features, which can subsequently be used by analytical tools to gather information required by the proposed study. Many algorithms have been proposed in the literature and the most common ones depend on the watershed transform, level-set theory, template matching, and wavelet analysis. For our pipeline the building block is the algorithm described in Al-Kofahi et al. (2010) which is a state-of-the-art algorithm for cell segmentation. This algorithm is based on a three step procedure. First, the image is divided into foreground and background regions using the graph-cuts algorithm. Second, cell centers (seed points) are found by a multi-scale Laplacian of Gaussian (LoG) filter constrained by the distance map. Third, the cells are reconstructed using a hill-climbing algorithm and then the shape is refined using the α-expansion algorithm. We have extended this algorithm to work on 16-bit/pixel images.

The above outlined algorithm becomes impractical if it is applied directly to images of the size required by the described study, since time and memory requirements grow exponentially. In addition, we need to quantify the presence of other bio-markers around the cells using secondary channels; this increases the memory requirements by a factor equal to the number of additional channels. For this reason, in our pipeline we have developed a divide-and-conquer method to correctly segment a montage of any size, using a selected tile size according to the processing capabilities of the system being used. The image montage is split into overlapping regions by using a big enough padding according to the maximum expected object size. Among the cells that lie on the border, some of them belong to the current tile and some others belong to the adjacent tile. When the adjacent tile is processed, these overlapping regions are segmented again. To merge the results and avoid object duplication, only the cells lying within the border are retained. All other cells on the border whose centers lie outside the actual tile are rejected. This approach was implemented in C++ using the Insight Toolkit, and it was parallelized using the OpenMP library to efficiently process multiple tiles simultaneously. The feature computation was also performed in parallel for each tile. These improvements in the algorithm implementation makes the use of the described algorithm practical for the problem at hand, something which was not possible before this pipeline was built. These features are subsequently used by the classification algorithm to distinguish between the different cell types present in the image. The most important cell type have been the microglia cells, since they are the driving hypothesis behind the failure of implanted neuro-prosthetic devices.

### 2.5. Cell type classification

One of the goals of this pipeline is to correctly identify cell types in multi-spectral images of brain tissue. This brain tissue comprises of cells of different types such as Neurons, microglia, Astrocytes, and Endothelial cells. An important issue concerning the electrode performance and the effects of device geometry is the proximity of these different cells to electrode sites. Thus, classification of these cells is a fundamental step in the analysis before characterizing their spatial distribution. In both these examples, the scale of the data being analyzed is extremely large. A typical dataset consists of hundreds of thousand of cells. This calls for a robust and efficient cell classification algorithm that is scalable to our needs, and which can be easily trained by a biologist. Human annotation is tedious, expensive and subjective. There will be intra- and inter-observer variance with respect to the selection of the most informative samples for the training of the classifier. In general, humans are biased at picking the most informative examples. For this reason, a mathematical tool which reduces this bias is required.

We have used a semi-supervised machine learning algorithm to train the classifier, which minimizes the amount of human effort. A special case of this kind of algorithms makes use of the active learning framework which essentially solves the problem of objectively picking the most informative set of examples from a large unlabeled pool; it is based on the assumption that not all training samples are equally informative. Active learning is a paradigm that helps in classifier learning with minimal effort from the user by concentrating on informative examples only. By querying the most informative examples at every iteration, we can build the classifier at a lower cost. Active learning methods are iterative and the algorithm updates its knowledge about the problem after obtaining the labels for the queried examples at each iteration. The active learning approach used in this pipeline is based on the logistic regression classifier as described by Padmanabhan (2012). As part of the development of this pipeline, this approach was integrated on the FARSIGHT software system, with an appropriate user interface to make it easy and intuitive for the biologist to train the classifier. However the large scale of the data makes it impractical to be used on a complete dataset. To solve this issue the pipeline was run on specific region of interest and the classifier was trained on these small regions which can be handled by the FARSIGHT user interface system. The designed classifier was later used on the full image montage. On average we have used 40 samples (iterations) per dataset to classify microglia with an overall accuracy of above 95% on all the datasets that we used in our proposed pipeline.

### 2.6. Tracing

After segmenting and classifying the cell nuclei of Microglia, the next step is to digitally reconstruct or trace the arbors of these tree like structures to extract meaningful information. These reconstructions form the basis for quantifying arbor morphologies for statistical analysis. Tracing algorithms can be classified based on the method used to digitally reconstruct these arbors. To our knowledge these algorithms can be classified into (i) Active Contour (Wang et al., 2011), (ii) Graph Based Methods (Xie et al., 2010), (iii) Minimal Path (Meijering, 2010), (iv) Sequential Tracing (Aylward and Bullitt, 2002), and (v) Skeletonization (Cohen et al., 1994). We have used graph based technique along with the minimal path methods to trace the arbors. The graph based tracing algorithm starts by collecting a number of initial points that lie along the center of the cell arbors. These interest points are referred to as Seed Points. Inaccurate detection of these seed points will lead to incorrect analysis. Most of the seed point detection algorithms are highly sensitive to the parameters and for our images, which are acquired under varying imaging conditions, we require algorithms that can learn from the images and which are robust to these varying imaging conditions.

We have developed a new tracing algorithm by integrating a widely used technique in the image processing literature, which consists of using appropriate dictionary learning of the image features. To the best of our knowledge, this is the first kind of tracing algorithm which uses this powerful mathematical technique in the field of bio-image analysis. Large-scale tracing of microglia arbors using over complete dictionaries (Megjhani et al., submitted) learns the curvilinear structure surrounding the putative seed points and classifies the structure based on the learned dictionary **D**. The algorithm extracts a block of size *x* × *y* × *z* surrounding the pixel and obtains the sparse features based on the sparse coding techniques using the dictionary **D**. Given the sparse features Γ and the classifier **L**, the next step is to classify the seed points based on the sparse representation of the image. The step to learn the classifier and the dictionary is given below

(3) $$\begin{array}{l} <D,L,\Gamma \geq {\text{arg min}}_{D,L,\Gamma }||Y-D\Gamma |{|}_{2}^{2}+\beta ||H-L\Gamma |{|}_{2}^{2},\\ \;s.t.\;\forall i,\;||\gamma_{i}|{|}_{0}⩽T \end{array}$$

where **Y** = {*y*_1_, *y*_2_, …, *y*_*N*_} is the matrix of size **R**^*n* × *N*^; and *n* = *x* × *y* × *z* and *N* is the total number of pixels in the image. **H** = {*h*_1_, *h*_2_, …, *h*_*N*_} ∈ **R**^*m* × *N*^ are the class labels for the input **X** for *m* classes, *m* in our case is 2, i.e., the pixel is either a seed point or not a seed point. The first term in (3) represents the squared reconstruction error. The second term in (3) represents the classification error for a weight matrix. The dictionary learned in this manner has excellent representational power, and enforces strong discrimination between the two classes (e.g., seed points and non-seed points). After learning the **D, L, Γ**; given a new image, the sparse representation Γ in **D** can be obtained using the sparse coding algorithms (Aharon et al., 2005), and given the sparse coding algorithm a pixel can be classified as a seed point by computing

(4) $$L\Gamma ={\left[l_{1},l_{2}\right]}^{T},$$

(5) $$S=\{\begin{array}{ll} 1\text{ or }l_{1}\text{ (Class 1 aka arbors)}, & \text{ if }l_{1}>l_{2}\\ 0\text{ or }l_{2}\text{ (Class 2 aka background)}, & \text{otherwise} \end{array}$$

where *S* has the collection of seed points. The next step after detecting the seed points is to determine how they are connected. We construct a Minimum Spanning Tree (MST) to model each microglia as described in Megjhani et al., (submitted); an MST like any graph consists of nodes and edges. In our case, each node is the location of pixels detected as seed points. Each edge is the cost of considering that a voxel belongs to the microglia process. The cost was defined by computing the geodesic distance between the two nodes. The MSTs were constructed using an adaptation of Prim algorithm Prim (1957). Starting from the root nodes, that are centroids of the microglia cell nuclei, the algorithm connects the closest primary nodes *S* in the sense of a geodesic metric. The detected link then seeks its nearest primary node to form the next link in the tree and thus the tree expands. The tree growing process runs in parallel for a given image and at the end of the tracing algorithm there are *K* MSTs where *K* is the number of Microglia cell nuclei present in the image. Applying this algorithm on an image containing few thousands of microglia becomes impractical due to the memory requirement. For this reason we have developed a dice-and-trace approach which divides the image into overlapping tiles centered at every microglia centroid. Each dice only has traces corresponding to one microglia cell. The dice size is selected according to the maximum expected arbor length of the microglia, and adjacent regions are included in order to accurately model the arbor growing process with respect to neighboring microglia cells. Each individual region is traced independently on a server with 40 cores (2 threads per core). The results are then merged together to create a final microglia morphology reconstruction of the whole image montage. The dice size is selected according to the maximum expected length of the microglia processes.

### 2.7. Visualization, clustering, and progression

The developed pipeline generates the results in a format which can be understood by the FARSIGHT system, an open source and cross-platform toolkit for analyzing multi-dimensional and multi-spectral biological images. Of particular interest for our project is to correctly visualize, edit, and analyze the segmentation and tracing results. Even the best available automated systems today have a non-zero error rate, implying the continued need for visual proofreading and corrective editing systems for which the FARSIGHT Trace Editor was used (Luisi et al., 2011). To group different types of Microglia for the analysis of distribution of the cells around the neuro-prosthetic device, we have used the clustering algorithm described in Lu et al. (2013). The Trace Editor system was optimized to efficiently edit the large dataset described in this study. The trace editor is developed in C++ and was integrated in the pipeline using the python language. This allowed us to efficiently separate the development of the user interface from the development of the main pipeline.

**Figure 4:**
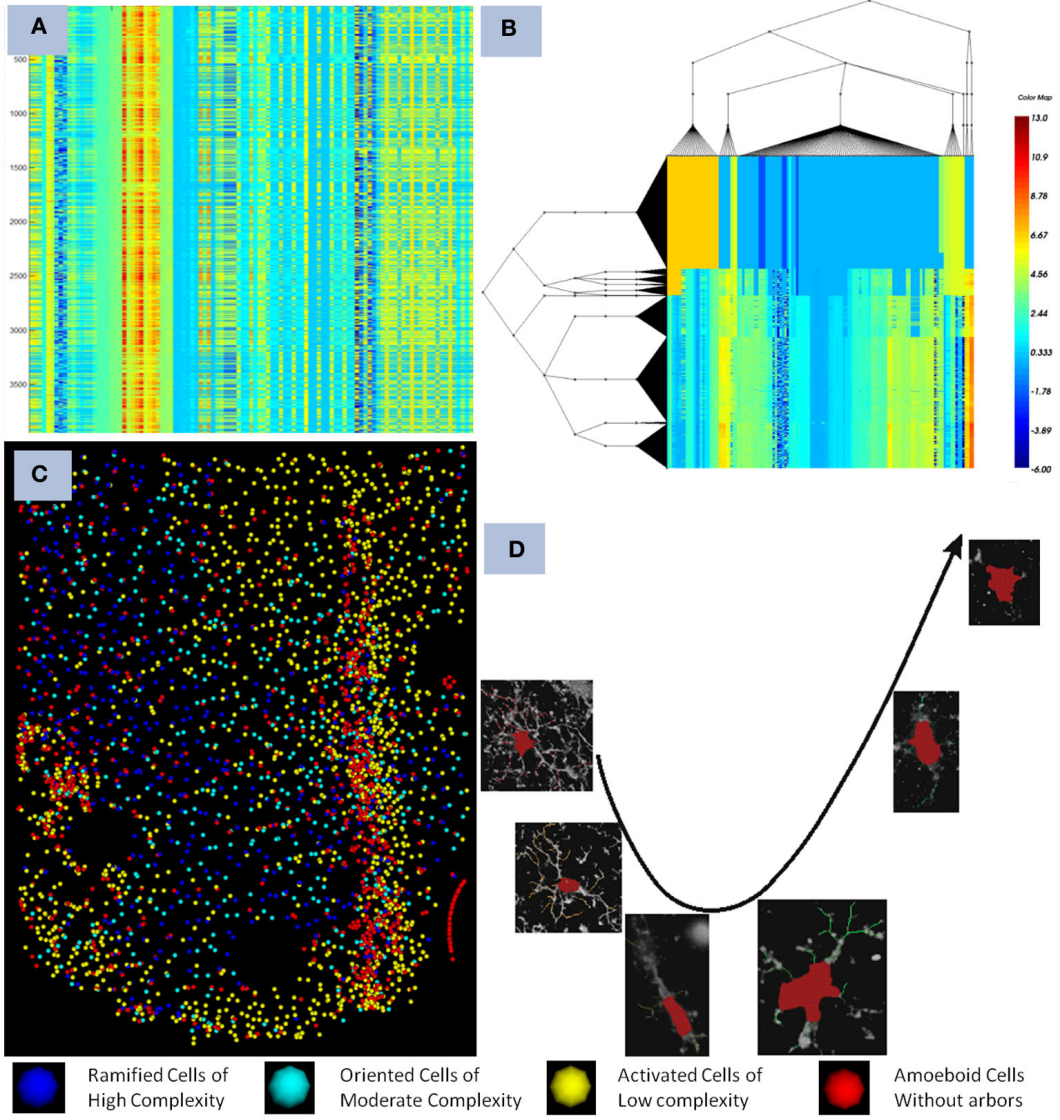
**Illustrates the final result obtained after the pipeline was run on a dataset containing a device. (A)** show the features computed for each microglia as seen in the heat map before clustering, each row corresponds to a cell, and each colum to a feature, **(B,C)** shows the co-clustering obtained displayed as a heat map and the corresponding distribution in the spatial domain with respect to the device, and **(D)** shows the progression of microglia states discovered using the method described in Xu et al. (2013). The pipeline creates an integration of all the modules together with the powerful visualization and analytical tools present in FARSIGHT.

The microglia cells are known to undergo cell arbor morphological changes in response to tissue perturbation. Ensembles of microglia exhibit a progression of arbor morphologies. It is of prime importance to study the progression or the spatial distribution of microglia arbor states. For this we have used Unsupervised Inference of Arbor Morphology Progression (Xu et al., 2013). Sample results of the clustering and progression algorithms are given in Figure 4. This algorithm was also integrated in our pipeline, and was also integrated in the FARSIGHT system. The integration of a methodology which is capable of offering the complete processing of such a complex image processing problem is what we consider our most important contribution to the image processing community.

### 2.8. Results

A total of 8 different datasets were processed with the developed pipeline. Figure 4 illustrates a summary of the results obtained after using the described pipeline. On average our approach takes 10 h to complete the entire process of image mosaicking, preprocessing, segmentation, cell classification, microglia tracing, and progression discovery on an image of 200 GB per channel, with 4 channels. The classification accuracy of the different cell types was above the 95% for all the datasets. The described piepline has a good integration with the visualization modules implemented into the FARSIGHT toolkit.

This pipeline was successfully used to generate the results presented in Lu et al. (2013), Xu et al. (2013). The developed pipeline enabled us to study how the microglia states affect the neuro-prosthetic device's capability to transmit signals. It was found that there is a clear progression of the microglia states as we move away from the device as shown in Figure 4.

### 2.9. Conclusions

The Python language is well-suited to implement a bioinformatics approach that encompasses a large number of interdependent steps, which are normally developed by independent groups to tackle specific problems. We found that the Python language was very well suited to our application, and it allowed us to integrate all the modules in order to obtain results with little or no effort. The series of modules covered in the analytical pipeline implemented in Python reflect the flexibility of this language to create a simple solution for an otherwise complex problem. The developed modular pipeline architecture was customized to perform the analysis of high-throughput high content brain tissue images. The pipeline was created with the idea of one-click automation which means that anyone can run the processing from start to finish with an intuitive user interface that allows the results to be visualized and edited.

The level of user input required to successfully operate the pipeline is reduced given that the combination of selected algorithms makes the process robust to changes in the input data. In some extreme cases, when the data changes significantly, a small amount of changes can be made in order to obtain a satisfactory solution. These changes are also easy to incorporate in the process given that the core building block is separated from the algorithms, by defining those parameters outside of the application. Results from the aforementioned pipeline were produced for more than 20 of such datasets. This pipeline was used by an extended group of people, who were able to contribute to specific parts of the inner modules/plugins. Without Python, the development process and integration of such vast number of modules would have been extremely difficult and time consuming given the fact that each group has a particular programming language preference.

In the future we will add the vessel channel to our pipeline. Vessels will help us add another layer of information to our analysis. This will increase the amount of information which can be used by our clustering and progression discovery algorithms in order to relate the underlying patterns when comparing normal with disturbed brain tissue. Finally, it is also important to highlight that all the code developed by this project is open-source and available trough the FARSIGHT repository at (http://farsight-toolkit.org/wiki/FARSIGHT_HowToBuild).

## Funding

This work was supported by DARPA Grant N66001-11-1-4015.

### Conflict of interest statement

The authors declare that the research was conducted in the absence of any commercial or financial relationships that could be construed as a potential conflict of interest.

## References

[B1] Aharon M. Elad M. Bruckstein A. (2005). K-svd: design of dictionaries for sparse representation, in Proceedings of SPARS'05, Workshop on Signal Processing with Adaptive Sparse/Structured Representations was held at IRISA, Rennes, France, 16–18 November 2005 (Rennes), 9–12

[B2] Al-Kofahi Y. Lassoued W. Lee W. Roysam B. (2010). Improved automatic detection and segmentation of cell nuclei in histopathology images. IEEE Trans. Biomed. Eng. 57, 841–852 10.1109/TBME.2009.2035102 19884070

[B3] Aylward S. R. Bullitt E. (2002). Initialization, noise, singularities, and scale in height ridge traversal for tubular object centerline extraction. IEEE Trans. Med. Imaging 21, 61–75 10.1109/42.993126 11929106

[B4] Candes E. Demanet L. Donoho D. Ying L. (2006). Fast discrete curvelet transforms. Multiscale Model. Simul. 5, 861–899 10.1137/05064182X

[B5] Cohen A. Roysam B. Turner J. (1994). Automated tracing and volume measurements of neurons from 3-d confocal fluorescence microscopy data. J. Microsc. 173, 103–114 10.1111/j.1365-2818.1994.tb03433.x 8169949

[B6] Ibanez L. Schroeder W. Ng L. Cates J. (2003). The ITK Software Guide, 1st Edn, Kitware, Inc. ISBN: 1-930934-10-6. Available online at: http://www.itk.org/ItkSoftwareGuide.pdf

[B7] Kelly B. McKay T. A. (2004). Morphological classification of galaxies by shapelet decomposition in the sloan digital sky survey. Astron. J. 127, 625 10.1086/380934

[B8] Lu Y. Trett K. Shain W. Carin L. Coifman R. Roysam B. (2013). Quantitative profiling of microglia populations using harmonic co-clustering of arbor morphology measurements, in Biomedical Imaging (ISBI), 2013 IEEE 10th International Symposium on (San Francisco, CA: IEEE), 1360–1363 10.1109/ISBI.2013.6556785

[B9] Luisi J. Narayanaswamy A. Galbreath Z. Roysam B. (2011). The farsight trace editor: an open source tool for 3-d inspection and efficient pattern analysis aided editing of automated neuronal reconstructions. Neuroinformatics 9, 305–315 10.1007/s12021-011-9115-0 21487683

[B10] Meijering E. (2010). Neuron tracing in perspective. Cytometry A 77, 693–704 10.1002/cyto.a.20895 20583273

[B11] Meijering E. H. Jacob M. Sarria J.-C. F. Unser M. (2003). A novel approach to neurite tracing in fluorescence microscopy images, in SIP (IEEE, Transactions on Biomedical Engineering), 491–495

[B12] Padmanabhan R. K. (2012). Active and Transfer Learning Methods for Computational Histology. PhD thesis, University of Houston, Houston, TX

[B13] Prim R. C. (1957). Shortest connection networks and some generalizations. Bell Syst. Tech. J. 36, 1389–1401 10.1002/j.1538-7305.1957.tb01515.x

[B14] Roysam B. (2013). The Farsight Toolkit. Available online at: http://farsight-toolkit.org/wiki/Main_Page

[B15] Schindelin J. Arganda-Carreras I. Frise E. Kaynig V. Longair M. Pietzsch T. (2012). Fiji: an open-source platform for biological-image analysis. Nat. Methods 9, 676–682 10.1038/nmeth.2019 22743772 PMC3855844

[B16] Tsai C.-L. Lister J. P. Bjornsson C. Smith K. Shain W. Barnes C. A. (2011). Robust, globally consistent and fully automatic multi-image registration and montage synthesis for 3-d multi-channel images. J. Microsc. 243, 154–171 10.1111/j.1365-2818.2011.03489.x 21361958 PMC3566673

[B17] Wang Y. Narayanaswamy A. Tsai C.-L. Roysam B. (2011). A broadly applicable 3-d neuron tracing method based on open-curve snake. Neuroinformatics 9, 193–217 10.1007/s12021-011-9110-5 21399937

[B18] Xie J. Zhao T. Lee T. Myers E. Peng H. (2010). Automatic neuron tracing in volumetric microscopy images with anisotropic path searching, in Medical Image Computing and Computer-Assisted Intervention-MICCAI 2010, 13th International Conference, Beijing, China, September 20–24, 2010, Proceedings, Part II (Springer Berlin Heidelberg), 472–479 10.1007/978-3-642-15745-5_58 20879349

[B19] Xu Y. Savelonas M. Qiu P. Trett K. Shain W. Roysam B. (2013). Unsupervised inference of arbor morphology progression for microglia from confocal microscope images, in Biomedical Imaging (ISBI), 2013 IEEE 10th International Symposium on (San Francisco, CA: IEEE), 1356–1359 10.1109/ISBI.2013.6556784

[B20] Yang G. Stewart C. V. Sofka M. Tsai C.-L. (2007). Alignment of challenging image pairs: refinement and region growing starting from a single keypoint correspondence. IEEE Trans. Patt. Anal. Mach. Intell. 23, 1973–1989 10.1109/TPAMI.2007.1116 17848778

